# Ephemeral But Influential? The Correlation between Facebook Stories Usage, Addiction, Narcissism, and Positive Affect

**DOI:** 10.3390/healthcare8040435

**Published:** 2020-10-26

**Authors:** Sen-Chi Yu, Hong-Ren Chen

**Affiliations:** 1 Department of Counseling and Applied Psychology, National Taichung University of Education, Taichung City 40306, Taiwan; 2 Department of Digital Content and Technology, National Taichung University of Education, Taichung City 40306, Taiwan; hrchen@mail.ntcu.edu.tw

**Keywords:** Facebook Stories, social media addiction, narcissism, positive affect

## Abstract

Despite the steep increase in Facebook Stories users, there is scant research on this topic. This study compared the associations of frequency of Stories update, frequency of news feed updates, time spent reading Stories, and time spent reading news feeds, with regard to social media addiction, narcissism, and positive affect in college students. We recruited a sample of 316 college students from Taiwan. The analytical results show that Facebook Stories are more addictive and provoke more positive affect than conventional news feeds. Moreover, only usage behaviors associated with Stories predict narcissism. This study also found that the prediction of news feeds with regard to addiction, narcissism, and positive affect also seems to be diminishing and is being replaced by those of Stories. Future studies on the psychological consequences and predictors of social media usage should regard Stories as a crucial variable.

## 1. Introduction

Facebook Stories are ephemeral, short user-generated photo and video collections that display shared content for a limited period of time [1]. Stories offer a news feed that relies on visual rather than written information. Ephemeral social media, such as Facebook/Instagram Stories or Snapchat, have become the primary usage behavior on social networking sites (SNSs) [1,2]. The growth rate of Facebook Stories since 2017 is over 800%, and it has more than 500 million users, making it an important way for people to connect with each other [2]. Despite the steep increase in Facebook Stories users, there is scant research on this topic.

With their unique functions, Facebook Stories have a unique charm for users [1,2,3]. First, Facebook Stories are ephemeral; if not specifically stored, the data disappear in 24 hours, and this characteristic makes readers more willing to read them first. Second, Facebook Stories can be given higher privacy settings. Third, Stories offer unique effects to enrich the images. The aspect ratio of the images is more oriented towards that of smartphone screens, thereby enhancing the immersion of users and usage pleasure. Fourth, the design of Facebook Stories can promote flow.

Whether Facebook Stories have different predictors or lead to different psychological consequences compared to conventional news feeds requires further investigation. Previous research has indicated that the frequency and duration of usage specific to Facebook are associated with SNS addiction [4]. Specifically, time spent browsing Facebook news feeds and chat rooms can predict SNS addiction [5,6]. A meta-analysis also found that Facebook logins, time spent on Facebook, and the number of Facebook friends correlate with SNS addiction [7]. However, Facebook Stories is a newer item and, unlike news feeds, relies on visual presentations, ephemeral novelty, and greater privacy. Whether these features make Facebook Stories more or less addictive warrants further research.

Research has shown that narcissism is correlated with SNS addiction [4,8,9]. The results of these studies have indicated that narcissists use SNSs to showcase themselves, attract the attention of others, and display their own capabilities and feelings of superiority. Narcissists have heightened needs for recognition and admiration [8]. SNSs provide narcissists with a stage on which they can seek to meet these needs. Compared to other Facebook functions, Stories enable users to draw even more attention to themselves. They are more likely to be clicked and read, and users can more easily obtain feedback from their Facebook friends through them. The numerous special effects stickers of Facebook Stories also capture more attention. However, little research exists on this function.

The types and strength of emotions elicited by Stories compared to traditional news feeds also require further investigation. Sagioglou and Greitemeyer’s study indicated that the emotions aroused by the use of Facebook depend on the meaningfulness of the activity [10]. Meaningless Facebook usage (such as aimless browsing) creates negative moods, whereas meaningful usage evokes positive moods. Burke and Kraut also pointed out that people derive benefits from online communication, as long as it comes from people they care about and has been tailored for them [11]. Compared to the readers of news feeds, which have looser privacy settings, the readers of Stories are generally closer friends. Bayer et al.’s study also discovered that interactions on ephemeral social media, such as Facebook/Instagram Stories or Snapchat, arouse more positive emotions than other media do and are second only to face-to-face contact [1]. This may be because Stories offer more diverse and interesting ways of presenting information (such as filters, various effects, and a polling mechanism for interactions with viewers). These functions enable users to draw more attention from their friends and make interactions more fun.

College students are an important user group of Facebook. Most college students have a Facebook account and they are also major users of Stories [12]. Bayer et al.’s study observed that college students have a preference for ephemeral forms of social media [1]. Punyanunt-Carter, Cruz, and Wrench further found that college students often prefer social interactions on SNSs over face-to-face interactions [13]. Therefore, in this paper, we compared the prediction of Facebook Stories with news feed with regard to SNS addiction, narcissism, and positive affect in college students.

## 2. Materials and Methods 

### 2.1. Participants and Procedures

Using convenient sampling, a total of 316 samples were obtained from five universities in Taiwan (mean age = 20.95, standard deviation (SD) = 2.70, females = 72.2%, males = 27.8%). A digital link to our questionnaire (via Google Forms) was sent to the students via the digital teaching platforms used in their courses. The introduction to the questionnaire explained our policy on privacy protection for all participants. Students who completed the questionnaire could enter a prize draw, and winners were awarded electronic gift certificates. No conflicts of interest were present in the current study. The Institutional Review Board of National Chung Cheng University granted Ethical approval to carry out the study (Ethical Application Ref: CCUREC108100801). All methods were performed in accordance with the relevant guidelines and regulations of the institution.

### 2.2. Measures

#### 2.2.1. Bergen Social Media Addiction Scale

We translated the Bergen Social Media Addiction Scale (BSMAS) into Mandarin Chinese to gauge the dependence of the participants on SNSs [14]. The BSMAS was modified from the Bergen Facebook Addiction Scale (BFAS) by replacing all mentions of “Facebook” with the more generalized term “social media” [15]. The BSMAS is a 6-item scale measuring the core elements of addiction (salience, mood modification, tolerance, withdrawal, conflict, and relapse). The Cronbach alpha of our BSMAS was 0.90, indicating good reliability.

#### 2.2.2. Narcissistic Personality Inventory–13 (NPI-13)

We translated the Narcissistic Personality Inventory–13 (NPI-13) into Mandarin Chinese [16]. NPI-13 is a 13-item shortened version of the 40-item Narcissistic Personality Inventory (NPI-40) [17]. The NPI-13 is a three-factor structure including Leadership/Authority (LA), Grandiose Exhibitionism (GE), and Entitlement/Exploitativeness (EE). NPI-13 has good reliability and convergent and discriminant validity [16]. There are two responses for each question item. The participants were asked to choose the one that better fit their own situation.

#### 2.2.3. PANAS-10

This scale was compiled to gauge the positive and negative emotions evoked by the use of social media. PANAS-10 is a 10-item shortened version of the 20-item Positive and Negative Affect Schedule (PANAS) [18]. In consideration of the emotional adjectives used in the literature regarding internet usage behavior, we selected 10 items from PANAS and added “When I use SNSs, I feel…” before each emotional adjective. The five positive affective states in PANAS-10 are interested, excited, enthusiastic, proud, and inspired. The five negative affective states are irritable, jittery, distressed, upset, and hostile. The Cronbach alphas of the PA and NA in this study were 0.91 and 0.93, respectively, both indicating good reliability.

## 3. Results

### 3.1. Preliminary Analyses

The participants had an average of 511.42 Facebook friends (SD = 388.67). On average, 5% of the participants did not use Facebook Stories, 74.4% of the participants read 1~10 Facebook Stories every day, 5.1% read 11~20 every day, 2.2% read 21~30 every day, and 13.3% read over 30 every day.

In contrast, 59.8% spent an average of less than 1 hour reading Facebook news feeds every day, 24.1% spent 1~2 hours, 5.1% spent more than 2 hours but less than 3 hours, and 11.1% spent 3 hours or more.

The primary factors promoting the use of Facebook Stories were as follows: Time sensitivity (30.9%), More diverse and interesting ways of presenting information (24.8%), Better privacy (18.8%), Ephemeral aesthetics (13.9%), Full screen visual effects (10.9%), and Various effects available (8.5%).

### 3.2. Statistical Analyses

We first conducted Pearson correlation analysis on Facebook usage behaviors (number of Facebook friends, time spent reading Facebook news feeds (TN), frequency of news feed updates (FN), time spent reading Facebook Stories (TS), and frequency of Facebook Stories updates (FS)) and potential psychological outcomes (the scores of BSMAS, NPI-13, and PANAS-10). Next, we eliminated the variables that did not present any significant correlations. Table 1 presents the analysis results. As can be seen, among all the Facebook usage behaviors, the correlation between the number of Facebook friends and potential psychological outcomes did not reach the level of significance or correlations were very low (e.g. less than 0.20). Likewise, the correlations between various NA and Facebook usage behaviors did not reach the level of significance. Thus, these two variables were eliminated.

We then used TN, TS, FN, and FS as the independent variables to predict social media addiction, narcissism, and positive affect. We used the MIMIC (multiple indicators multiple causes) model instead of traditional regression to analyze the relationships between IVs and DVs. The MIMIC is a structural equation modeling-based technique that involves latent variables that are predicted by observed variables [19]. This model has three major advantages over traditional regression analysis: (1) evaluation of the confirmatory factor model on the latent variables and adjustment of obtained estimates for the effects of all the covariates in the model; (2) assessment of measurement errors; (3) model testing and assessed data-model fit [19,20,21]. The results of the MIMIC models were described as follows.

#### 3.2.1. Model 1: MIMIC Model on BSMAS

**Figure 1:**
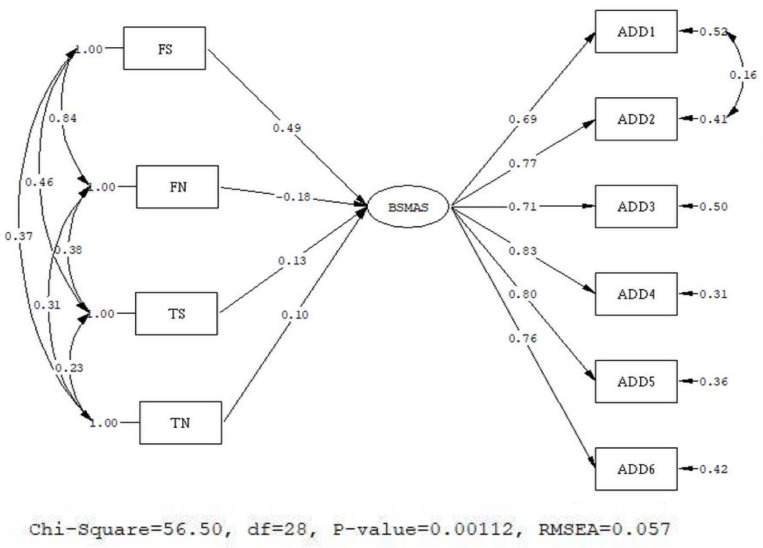
Multiple indicators multiple causes (MIMIC) model of the Bergen Social Media Addiction Scale (BSMAS). FS—frequency of Facebook Stories updates; FN—frequency of news feed updates; TS—time spent reading Facebook Stories; TN—time spent reading Facebook news feeds.

The original model showed a mediocre fit (Chi-Square = 92.68, df = 29, Root mean square error of approximation (RMSEA) = 0.083, 90% CI for RMSEA = (0.065; 0.100), Comparative Fit. Index (CFI) = 0.98, Standardized Root Mean Square Residual (SRMR) = 0.033). The modification index (MI) provided by statistical software (LISREL 8.80) indicates that error covariance should be added between items 1 and 2. Correlated errors indicate that items are highly correlated and those unique variances of the associated indicators overlap. Correlated errors are commonly used in model re-specification strategies of SEM methodology [19]. Since item 1 (You spend a lot of time thinking about social media or planning how to use it) and item 2 (You feel an urge to use social media more and more) are related in meaning, we added correlated errors between these two items. The revised model showed good fit (χ^2^ = 56.50, df = 28, RMSEA = 0.057, 90 % CI for RMSEA = (0.035; 0.078), CFI = 0.97, SRMR = 0. 028). The results showing the standardized solution of SEM are presented in Figure 1.

We used scores of BSMAS as the latent dependent variables; the standardized coefficients of FS, TS, FN, and TN are 0.49 (*p* < 0.05), 0.13 (*p* < 0.05), −0.18 (*p* > 0.05), and 0.10 (*p* > 0.05), respectively. These results indicate that FS has the greatest predictive coefficient. Furthermore, only usage behaviors associated with Stories predict social media addiction significantly. The prediction coefficients of usage behaviors associated with news feeds did not reach the level of significance. We believe that a high degree of co-linearity exists between the usage behaviors of Facebook Stories and those of news feeds, which led to the standardized coefficients of usage behaviors associated with news feeds not reaching the level of significance.

#### 3.2.2. Model 2: MIMIC Model on NPI-13

**Figure 2:**
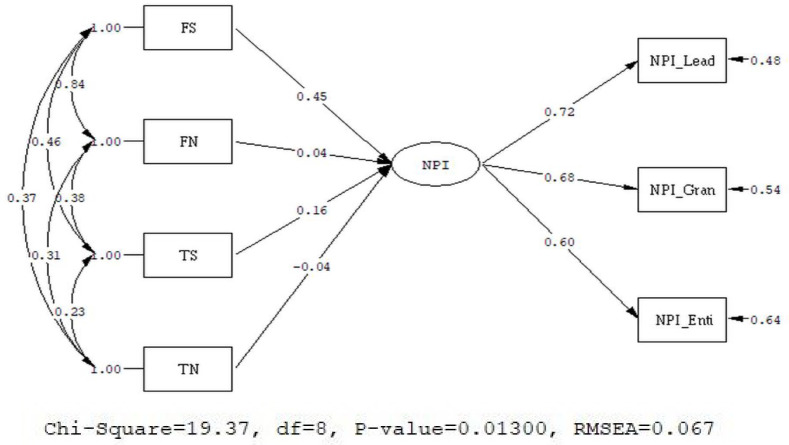
MIMIC model of Narcissistic Personality Inventory–13 (NPI-13).

The model, as presented in Figure 2, showed a good fit (χ^2^ = 19.37, df = 8, RMSEA = 0.067, 90% CI for RMSEA = (0.029; 0.11), CFI = 0.99, SRMR = 0.031). 

We used the scores of NPI-13 as the latent dependent variables; the standardized coefficients of FS, FN, TS, and TN are 0.45 (*p* < 0.05), 0.04 (*p* > 0.05), 0.16 (*p* < 0.05), and −0.004 (*p* > 0.05), respectively. These results indicate that FS has the greatest prediction. Furthermore, only usage behaviors associated with Stories can predict narcissism. The prediction coefficients of usage behaviors associated with news feeds did not reach the level of significance.

#### 3.2.3. Model 3: MIMIC Model on PA

**Figure 3:**
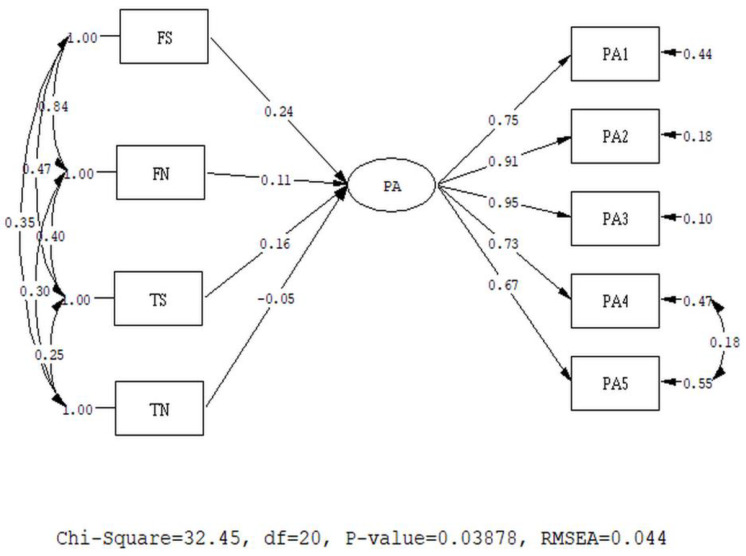
MIMIC model of Positive Affect Schedule (PA).

The original model showed a mediocre fit (χ^2^ = 77.02, df = 21, RMSEA = 0.092, 90% CI for RMSEA = (0.071; 0.11), CFI = 0.98, SRMR = 0.048). The modification index (MI) indicates that error covariance should be added between items 4 (proud) and 5 (inspired). We added correlated errors between these two items. The revised model, as presented in Figure 3, showed a good fit (χ^2^ = 56.50, df = 28, RMSEA = 0.057 with 90% CI (0.010; 0.071), CFI = 0.99, SRMR = 0.045)

The standardized coefficients of FS, FN, TS, and TN are 0.24 (*p* < 0.05), 0.11 (*p* > 0.05), 0.16 (*p* < 0.05), and −0.005 (*p* > 0.05), respectively. These results indicate that FS has the greatest prediction. Furthermore, only usage behaviors associated with Stories predict positive affect. The prediction coefficients of usage behaviors associated with news feeds did not reach the level of significance.

## 4. Discussion

This study discovered that some Facebook usage behaviors are associated with social media addiction. This confirms the findings derived by Muench, Hayes, Kuerbis, and Shao and Frost and Rickwood [6,7]. However, the Facebook usage behaviors in these two studies only included frequency of Facebook logins, time spent on Facebook, and daily time spent on Facebook. They did not examine the connection between Stories usage behavior and social media addiction.

This study found that Stories usage behaviors have greater prediction with regard to social media addiction than news feeds usage behaviors. This indicates that Stories may be more addictive than news feeds. The researchers believe that the characteristics of Stories can be used to explain this result. (1) Stories offers higher privacy settings, so they may only be shared with closer friends, and responses from these friends are more highly anticipated. (2) Stories are ephemeral and generally only last for 24 h. As a result, whomever they are shared with will likely read them first, and the posters will more likely make time to read the responses. (3) News feeds often contain too many irrelevant ads and news from less intimate friends. In contrast, the content of posted Stories is simpler; they exist to share news and links with close friends. (4) Facebook Stories offer unique effects to enrich posted images. The aspect ratio of the images is oriented towards that of smartphone screens, thereby enhancing the immersion of users and usage pleasure. With these features, users are more inclined to keep reading and posting on Stories, making them more addictive than news feeds.

This study found a correlation between Facebook usage and narcissism, which supports the findings derived by Andreassen, Pallesen, and Griffiths; Lim; Kuss and Griffiths; Sheldon and Bryant, 2016; Sheldon, Rauschnabel, and Honeycutt [8,14,22,23,24]. We also discovered that Stories usage behaviors have greater prediction with regard to narcissism than news feeds usage behaviors. Sheldon and Bryant [25] also pointed that some psychological circumstances, such as narcissism, may reinforce different behavioral tendencies and trigger particular SNS usage (i.e., editing photos, using hashtags). We believe that one of the reasons for this is that narcissists have strong needs for recognition and admiration [8]. Stories provides a stage for recognition and admiration, and compared to news feeds, the ephemeral characteristic of Stories makes it easier for narcissists to attract the attention of others and receive immediate responses. Narcissists are thus more likely to post information in their Stories to obtain attention and feedback, and the feedback or likes received provide narcissists with rewards and recognition [14]. Narcissists use social media not to establish social relationships but for the sake of self-exhibition [22,23,24]. In addition, narcissists use the positive feedback from interaction partners to regulate their self-esteem [25]. This shows that Stories provides narcissists with a new stage for self-exhibition and self-regulation.

This study found a correlation between Facebook usage and positive affect, which is consistent with the results obtained by Burke and Kraut [11]. Bayer et al.’s study also pointed out that ephemeral communication methods are intriguing and provoke more positive emotions [1]. We further found that Stories usage behaviors have greater prediction with regard to positive affect than news feeds usage behaviors. This means that the contents of Stories can provoke positive emotions better than news feeds can and we infer that this is because Stories are more customized. Moreover, users often only share them with closer friends, which promote a better sense of well-being and greater positive emotions [26]. Burke and Kraut’s study discovered that when people interact with close friends online and the content of the interactions are tailored to them, they feel happier than if they interact with general acquaintances [11]. Punyanunt-Carter, Cruz, and Wrench’s study also found that compared to other common SNS platforms, SNSs with ephemeral interactions are considered to be more interesting and provoke more positive emotions [13]. 

In conclusion, Stories are more appealing to users, provoke more positive affect, are more addictive, and are more likely to become stages where narcissists can showcase themselves and draw the attention of others than conventional news feeds. This study also found that Stories usage is a more significant determinant of psychological consequences than conventional news feeds usage. The prediction of news feeds with regard to addiction, narcissism, and positive affect also seems to be diminishing and is being replaced by those of Stories. Future studies on the psychological consequences and predictors of social media usage should regard Stories as a crucial variable.

This study has the following limitations. First, due to the descriptive nature of this study, we cannot identify the potential causes behind the described phenomenon. Future studies should consider experimental design to better understand the possible cause and effect relationships of the variables discussed. 

Second, this study used a web-based and non-random sampling. Although this sampling method is suitable for the purpose of this study (usages of social network sites) and target population (college students), generalizations cannot be made about the whole population. Besides, the participants of this study comprised far more women than men, which may cause inferential bias.

Third, concerning cultural generalizability, this study employed a single sample of Taiwanese college students. Further studies could attempt to sample different cultural groups, to establish generalizability. 

Fourth, concerning the applications, investigations on Stories usage should be expanded. For instance, Instagram is a popular app among younger generations, so future studies should consider the possible predictors and relevant factors of Instagram usage.

## 5. Conclusions

This study compared the associations of Facebook usages (frequency of Stories update, frequency of news-feed updates, time spent reading Stories, and time spent reading news feeds) and potential psychological outcomes (social media addiction, narcissism, and positive affect) in college students. We found Facebook Stories-related usages were better predictors than conventional related usages. Facebook Stories are more addictive and trigger more positive affect and narcissism. The influence of Facebook Stories must not be overlooked in future studies.

## Figures and Tables

**Table 1:** Correlations for scores of NPI-13, PANAS, BSMAS and Social Media Use.

Measure	1	2	3	4	5	6	7	8	9
1. NF	−	−0.030	0.002	−0.090	−0.030	0.136 *	0.085	0.108	0.190 *
2. FS		−	0.835 **	0.461 **	0.361 **	0.467 **	0.395 **	−0.056	0.404 **
3. FN			−	0.382 **	0.303 **	0.399 **	0.374 **	−0.048	0.298 **
4. TS				−	0.229 **	0.317 **	0.299 **	0.033	0.279 **
5. TN					−	0.148 *	0.122 *	−0.004	0.230 **
6. NPI-13						−	0.377 **	−0.095	0.268 **
7. PA							−	0.046	0.472 **
8. NA								−	0.250 **
9. BSMAS									−

Note: NF—number of Facebook friends; FS—frequency of Facebook Stories updates; FN—frequency of news feed updates; TS—time spent reading Facebook Stories; TN—time spent reading Facebook news feeds. NPI-13—Narcissistic Personality Inventory–13; PA—Positive Affect Schedule; NA—Negative Affect Schedule; BSMAS—Bergen Social Media Addiction Scale; * *p* < 0.05; ** *p* < 0.01.
